# Do breast cancer patients have a gender preference when choosing a breast surgeon?

**DOI:** 10.3332/ecancer.2023.1574

**Published:** 2023-07-14

**Authors:** Josepmilly del Valle Peña Colmenares, Yazmin José Velásquez Velásquez, Wladimir José Villegas Rodríguez, Leider Arelis Campos Pino, Álvaro Gómez Rodríguez, Douglas José Angulo Herrera

**Affiliations:** 1 Breast Pathology Service, Hospital Oncology Department (SOH), Venezuelan Institute of Social Security, Caracas 1040, Venezuela; 2 School of Statistics and Actuarial Sciences, Central University of Venezuela, Caracas 1053, Venezuela; a https://orcid.org/0000-0002-1114-6289; b https://orcid.org/0000-0003-3307-2564; c https://orcid.org/0000-0001-8999-9751; d https://orcid.org/0000-0002-0907-8467; e https://orcid.org/0000-0003-3740-0238; f https://orcid.org/0009-0003-5506-0297

**Keywords:** gender, breast cancer, surgeon, breast

## Abstract

**Introduction:**

There is a paucity of data on breast cancer (BC) patients’ gender preferences when it comes to choosing their surgeon, unlike in other specialties like gynaecology and obstetrics, where women tend to prefer a female physician. The aim of this trial was to examine if there are any gender preferences in women with BC at the time of choosing their breast surgeon.

**Material and methods:**

A cross-sectional, observational and descriptive study with 528 patients, older than 18 years, at the Breast Department ‘Servicio Oncológico Hospitalario del Instituto Venezolano de los Seguros Sociales’, from January to June 2022. We applied an anonymous questionnaire to evaluate patients’ gender preferences when it comes to choosing their breast surgeon.

**Results:**

The average age of the patients was 56 ± 11 years. 89.4% did not have gender preferences, whereas 6.5% and 4.1% chose to be treated by female surgeons and male surgeons, respectively. The most important characteristics chosen by the patients when they decided to choose their breast surgeon were experience (75%), knowledge (54%) and hospital-based (41%).

**Conclusion:**

Personal and professional skills are the most important factors when it comes to patients choosing their breast surgeon, gender does not have any impact on expertise or competence.

## Introduction

The doctor-patient relationship and the growing autonomy of patients in choosing their health service providers play a fundamental role in health care [1]. It is thus notable that few studies have examined the importance of doctor gender when patients choose a breast surgeon. But it seems patients tend to prefer female care providers when considering more intimate clinical scenarios, such as physical exams in obstetrics and gynaecology [2–5].

Patient preferences for doctor gender is a perception issue, especially in the context of surgical treatment of breast cancer (BC). Previous studies showed that patients often prefer female providers [4, 5] but did not determine whether patients have gender preferences for breast surgeons. The study conducted by Hsu *et al* [1] used 1,413 surveys to determine social preferences for the gender of breast surgeons and cosmetic and reconstructive surgeons. That survey posed a hypothetical scenario: patients required a mastectomy and then wanted breast reconstruction. The study found 57% and 53% of respondents, respectively, had no gender preference for their breast surgeon or their plastic and reconstructive surgeon. But 40% of respondents preferred that a female surgeon perform a mastectomy.

One of the few studies on this topic, published in the late 1990s by Reid et al [3], aimed to determine the preference of women for the gender of their breast surgeon. That study, conducted via a survey in the UK in 1997, found that roughly one-third of women preferred referrals to a breast clinic with a female surgeon. However, in 1996, only 2.3% of general surgeons in England and Wales were women [3]. Circumstances have changed, with more women pursuing surgical specialties; and patients have perceptions, beliefs, customs and/or traditions that may influence their gender preference at the point of care.

The results of that 1998 study are thus an initial reference for the purpose of our research: to determine whether women with BC have a gender preference when choosing a surgeon in the Breast Pathology Service in the Hospital Oncology Department [SOH (Servicio Oncológico Hospitalario)], or if the personal and professional skills of surgeons influence our patients when they make this decision.

### Objectives

#### Primary objective

The primary objective of this study was to determine whether women have gender preferences when choosing a breast surgeon.

#### Secondary objective

The secondary objective was to determine the most important characteristics patients consider when choosing a breast surgeon.

#### Materials and methods

A cross-sectional, observational and descriptive study of 582 patients 18 years or older who came to the Breast Pathology Service at the SOH-IVSS (Servicio Oncológico Hospitalario del Instituto Venezolano de los Seguros Sociales) a public institution and one of three public centres specialising in cancer care in Venezuela.

The study sample was intentional due to the absence of an estimator to calculate the sample size. An anonymous survey was used to evaluate the preferences of patients when choosing their breast surgeon. Six hundred and forty patients were surveyed and 582 were selected after excluding those who completed surveys incorrectly. These patients came to the Breast Pathology Service on consecutive Wednesdays and Thursdays between 15 January and 15 June 2022. The hospital Ethics Committee reviewed and approved the study protocol.

The survey comprised 16 items, the first part including patient demographic information, religion, marital status and education level. The second part asked patients to review a list of 14 traits and select the 3 most important ones for choosing a breast surgeon. The last part of the survey included questions about gender preference in surgical medical practice, such as physical exams of the breasts and surgeries. Finally, the survey included questions about more subjective perceptions, such as the personality of the doctor and/or the doctor-patient relationship.

### Statistical methods

The arithmetic mean and standard deviation for age and the frequencies and percentages of other variables were calculated. A chi-squared test was used, and a dichotomous logistical regression model was applied. The differences between variables by evaluated domain were calculated using the marginal homogeneity test. The differences between epidemiological variables by characteristics were calculated using Pearson’s chi-squared test. The data were tabulated using SPSS 26. A *p* value <0.05 was considered significant.

## Results

The study population included 582 of 640 patients with BC who completed surveys about their gender preference when choosing a breast surgeon. The median patient age was 56 ± 11 years. Most patients, 89.4%, had no gender preference, whilst 6.5% and 4.1% preferred female and male surgeons, respectively. Bivariate analysis of demographic characteristics showed that religion was the sole statistically significant variable (*p* = 0.030). The proportion of Catholic women who preferred male surgeons was less than those who preferred female surgeons or had no preference. Results for education level showed that most patients had no gender preference, though patients who chose a female surgeon were university graduates. But Table 1 shows there were no significant differences.

Table 2 shows that of the three most important traits patients selected when choosing a breast pathology surgeon, 75% and 54% of patients selected experience and knowledge, respectively. Other variables that patients listed as preferring were working in hospitals (41%), availability (39%) and skill (28%).

Results for clinical-surgical practice preferences (breast exams and breast surgeries) showed that under 10% of patients chose a male or female surgeon. Only 6% of patients chose female surgeons for physical breast exams, and over 89% had no gender preference for this variable (Table 3).

Embarrassment during the exam was not a significant reason for preferring a surgeon of the same sex, and only 12% of patients felt embarrassed when male surgeons examined them. We also found no preferences for psychosocial factors corresponding to the personality and professionalism of a doctor when choosing a breast surgeon. But there were significant differences between three variables: feelings during physical exams (*p* = 0.002), the personality (*p* = 0.024), and professionalism of the doctor (*p* = 0.007) (Table 4).

A multivariate regression model was completed to determine odds ratios for preferring female surgeons over male surgeons and having no preference. No statistical association was observed between variables, meaning that age, religion, marital status and university education are independent variables in the choice of a female or male surgeon (Table 5).

## Discussion

Opinion in Venezuela has held that female patients, especially those with breast diseases, preferred female surgeons, but this belief had never been shown scientifically through descriptive research. Nonetheless, data keep evolving despite the scarcity of published studies on this topic in Venezuela and across Latin America. Ethnicity, religion or certain demographic characteristics probably influence patients when they decide to seek a consultation or treatment for any breast disease. This study found no differences in the results of patient-reported satisfaction with surgeons.

But there are multiple studies of gender preference in other specialties. For example, five studies of obstetrics and gynaecology in the United States found that 27.6%–52.8% of patients preferred a female doctor [4–8]. Dineen *et al* [9] showed that most patients (83.9%) had no gender preference when choosing an orthopaedic surgeon. Only 14.5% of patients preferred female surgeons, and even this preference varied by subspecialty.

Notably, in one of the largest studies conducted in the United States; which used surveys on surgeon gender in a hypothetical, clinical BC scenario; 40% of women respondents reported preferring a female breast surgeon. Our survey also asked respondents why they preferred one gender of the surgeon over the other. Most respondents mentioned listening, empathy, understanding and communication as reasons for their preference; they also reported reasons related to comfort [1].

Moreover, Bertakis [10] noted that female doctors showed different approaches to medicine. For example, they were more interested in knowing the feelings of their patients and reaching an agreement with them when making decisions. These practice styles have been described as ‘feminine traits’.

An Israeli study showed that two-thirds of breast clinic patients had no gender preference for surgeons, while one-third preferred only female breast surgeons [2]. A review of aesthetic plastic surgery [11] found comparable results: 46% of women respondents had no gender preference. In contrast, the reviews by Bashour and Abdulsalam [7] and Lafta [8] found that 85% of Syrians and 74% of Iraqi women preferred female gynaecologists, showing an association between gender, social tradition and religious beliefs.

One of the few studies published on gender preference for breast surgeons was conducted in the 1990s (1997) in London. That study used surveys and found that 31% of respondents preferred female surgeons [3]. In the Israeli report, 28.2% of the patients preferred female surgeons [2], much higher than in our study, which found only 6.5% of patients chose a female breast surgeon. We do not know if cultural differences and religious beliefs could have influenced our results. Our study population was predominantly Catholic, unlike the populations of the aforementioned studies that have different cultural traditions.

Our study also evaluated preference in surgical clinical practice, specifically physical examinations, which are considered a more personal and intimate procedure. The study by Groutz *et al* [2] found that one-third of patients preferred a female breast surgeon (32%) during the physical examination, but not for surgery. In our study, only 6% of patients preferred to be evaluated (physical examination) by female surgeons. But we found patients had no preference when choosing a surgeon to perform an invasive breast surgery, which patients may see as a potential threat to their health. This finding implies that patients intrinsically value the professional skills of surgeons more than their gender.

Most patients diagnosed with BC (89.4%) did not have a gender preference when choosing a surgeon. That finding was comparable to those from the studies by Reid [3] and Hsu *et al* [1] but contrasted with other studies that found women prefer female obstetricians and gynaecologists [12, 13].

Patients in our study do not think the sex of surgeons affects their skills. The determining factor patients consider in surgical treatment is having good surgeons with experience and expertise who work in public hospitals. But we cannot rule out that this last factor resulted from conducting the survey in a (public) cancer hospital. As in other studies, the experience, surgical skill, knowledge and reputation of the doctor outweighed gender [2, 6, 9, 14].

Various reviews have evaluated the gender biases and stereotypes of male and female surgeons in general surgery and plastic surgery [15, 16]. Our study used binary gender categories.

Finally, we reiterate that there are no publications in Venezuela concerning gender preference when choosing a breast surgeon. But we know more studies are needed, not only in Venezuela, but across Latin America, to determine if gender preferences play an important role in choosing service providers for breast disease.

### Observations

Our data cannot be generalised because our sample is from a single public hospital, though the SOH-IVSS Breast Pathology Service is a national reference centre for BC treatment. The basis of our study was a survey of patients who sought care for breast disease at this institution, and we therefore cannot rule out percentage bias in the views collected for this variable. But notably, this is the only study of this topic conducted in Venezuela. It would thus be desirable for a specialised, private breast pathology centre to conduct a similar study and then compare the results obtained from public and private sector patients.

### Practice implications

Our study starts a line of Venezuelan research that aims to determine the gender of both surgeons treating BC and other specialists using intimate clinical scenarios. This research should include patients in various healthcare settings, especially public ones, as study results may improve services by considering patient preferences for the gender of their providers.

By producing relevant results, this line of research could make it possible to conduct similar studies in other public and private centres that specialise in treating breast diseases. Future studies could examine other independent variables besides the male/female gender divide, including religion, socioeconomic conditions and culture.

## Conclusion

Personal and professional skills were the most important factors when patients in our study sample chose the sex of a breast surgeon. The gender of surgeons does not affect their expertise or skill, because as Moraila *et al* [17] noted, ‘talent is a universal value in humans, regardless of gender’.

## Conflicts of interest

The authors declare that they have no conflicts of interest that could have affected the results of the study.

## Funding

No financial support was received for writing this article, for data collection or analysis or for preparing or approving this current manuscript.

## Disclosure

The authors have no financial interest to declare related to the disclosure of the content of this article. Their only interest focuses on opening a line of research on the topic of the study in Venezuela.

## Figures and Tables

**Table 1.** General characteristics of study patient participants SOH-IVSS (January–June 2022).

		Response on preference							
		Indifferent		Prefers female		Prefers male			
		520		38		24			
Variables	Categories	*n*	%	*n*	%	*n*	%	Total	*p*
Age	≥45 years	441	84.8	29	76.3	21	87.5	491	0.35
	<45 years	79	15.2	9	23.7	3	12.5	91	
Religion	Catholic	507	97.5	38	100.0	21	87.5	566	0.03
	Jewish	1	0.2	0	0.0	0	0.0	1	
	Other	12	2.3	0	0.0	3	12.5	15	
Religious	Yes	494	95.0	36	94.7	22	91.7	552	0.77
	No	26	5.0	2	5.3	2	8.3	30	
Marital status	Single	259	49.8	18	47.4	15	62.5	292	0.59
	Married	169	32.5	13	34.2	4	16.7	186	
	Other	92	17.7	7	18.4	5	20.8	104	
Level of education	No studies	7	1.3	1	2.6	1	4.2	9	0.27
	Primary education	129	24.8	9	23.7	2	8.3	140	
	Secondary education	156	30.0	10	26.3	12	50.0	178	
	TSU	62	11.9	7	18.4	1	4.2	70	
	University	166	31.9	11	28.9	8	33.3	185	


Higher National Certificate (TSU)

Source: SOH-IVSS Breast Pathology Service surveys

**Table 2.** Characteristics selected by patients as important when choosing a breast surgeon.

Important features	Variables	*n*	%
Demographics	Age	26	4.5
	Gender	9	1.5
	Origin	3	0.5
	Marital status	3	0.5
	Religious status	7	1.2
Professional skills	Ability	163	28.0
	Experience	434	74.6
	Knowledge	316	54.3
Academic merits	Have attended university	53	9.1
	Work in hospitals	241	41.4
	University affiliation	52	8.9
Other qualities	Personality	114	19.6
	Reputation	100	17.2
	Availability	229	39.3

Source: SOH-IVSS Breast Pathology Service surveys

**Table 3.** Patient preference in choosing service providers in clinical–surgical practice.

	Indifferent		Prefers female		Prefers male		
Variables	*n*	%	*n*	%	*n*	%	Total
Breast examination	**526**	**90.4**	**35**	**6.0**	**21**	**3.6**	**582**
Physical examination	**521**	**89.5**	**42**	**7.2**	**19**	**3.3**	**582**
Breast surgery	**520**	**89.3**	**38**	**6.5**	**24**	**4.1**	**582**
Other surgeries	**543**	**93.3**	**20**	**3.4**	**19**	**3.3**	**582**

Multiple comparisons: *p* = 0.031

**Table 4.** Psychosocial aspects of gender preference of patients in the choice of breast surgeon.

		Indifferent		Prefers female		Prefers male			
Variables	Answers	*n*	%	*n*	%	*n*	%	Total	*p*
Feeling during breast examination	More shame	496	85.2	17	2.9	69	11.9	582	0.002
	More comfortable	436	75.0	119	20.4	27	4.6	582	
	Softer	476	81.8	71	12.2	35	6.0	582	
Physician’s personality	More understanding	428	73.5	114	19.6	40	6.9	582	0.024
	More patient	452	78.0	82	14.1	48	8.2	582	
	More time with the patient	459	78.8	76	13.1	47	8.1	582	
More medical professionalism	More understanding	443	76.1	109	18.7	30	5.2	582	0.007
	More knowledgeable about women's health	438	75.2	107	18.4	37	6.4	582	
	Best overall physician	493	84.7	58	10.0	31	5.3	582	

Differences between variables by domains: Feeling during breast examination; Physician’s personality: *p* = 0.024

Source: SOH-IVSS Breast Pathology Service surveys

**Table 5.** Odds ratios associated with care preferences of women over men and without preferences for women versus men.

Variables	Answers	OR	IC-95%		*p*
Female preference over male	Age ≥ 45 years old	0.46	0.11	1.91	0.45
	Catholic religion	0.36	0.25	0.50	0.10
	Married	2.60	0.73	9.22	0.22
	University education	0.82	0.27	2.45	0.94
No preferences over male or female	Age ≥ 45 years old	1.34	0.68	2.63	0.50
	Catholic religion	1.98	0.55	7.16	0.51
	Married	1.28	0.71	2.29	0.51
	University education	1.06	0.60	1.88	0.95

Chi-squared test and the dichotomous logistic regression model were applied

Source: SOH-IVSS Breast Pathology Service surveys
